# A Fragment-Based Screen for Inhibitors of Escherichia coli N5-CAIR Mutase

**DOI:** 10.21203/rs.3.rs-4921418/v1

**Published:** 2024-09-20

**Authors:** Marcella F. Sharma, Steven Firestine

**Affiliations:** Wayne State University Eugene Applebaum College of Pharmacy and Health Sciences; Wayne State University Eugene Applebaum College of Pharmacy and Health Sciences

**Keywords:** N5-CAIR mutase, screening, fragment, thermal melting, enzyme inhibitors

## Abstract

Although purine biosynthesis is a primary metabolic pathway, there are fundamental differences between how purines are synthesized in microbes versus humans. In humans, the purine intermediate, 4- carboxy-5-aminoimidazole ribonucleotide (CAIR) is directly synthesized from 5-aminoimidazole ribonucleotide (AIR) and carbon dioxide by the enzyme AIR carboxylase. In bacteria, yeast and fungi, CAIR is synthesized from AIR via an intermediate N^5^-carboxyaminoimidazole ribonucleotide (N^5^-CAIR) by the enzyme N^5^-CAIR mutase. The difference in pathways between humans and microbes indicate that N^5^-CAIR mutase is a potential antimicrobial drug target. To identify inhibitors of *E. coli* N^5^-CAIR mutase, a fragment-based screening campaign was conducted using a thermal shift assay and a library of 4,500 fragments. Twenty-eight fragments were initially identified that displayed dose-dependent binding to N^5^-CAIR mutase with K_d_ values ranging from 9–309 μM. Of the 28, 14 were obtained from commercial sources for retesting; however, only 5 showed dose-dependent binding to N^5^-CAIR mutase. The five fragments were assessed for their ability to inhibit enzyme activity. Four out of the 5 showed inhibition with K_i_ values of 4.8 to 159 μM. All fragments contained nitrogen heterocycles with 3 out of the 4 containing 5-membered heterocycles like those found in the substrate of the enzyme. The identified fragments show similarities to compounds identified from studies on *B. anthracis* N^5^-CAIR synthetase and human AIR carboxylase suggesting a common pharmacophore.

## Introduction

Purines are heterocyclic aromatic compounds containing a 5,6-fused ring system. They are key components of nucleic acids, energy carriers, and signal transduction pathways [1]. As such, the synthesis of purines is critical for the survival of organisms. Purines are made by one of two methods: the *de novo* or the salvage pathway. The salvage pathway recycles nucleotides generated from the breakdown of nucleic acids [2]. This pathway maintains constant concentrations of purine nucleotides in cells; however, the salvage pathway produces less than 1% of the needed nucleotides for DNA synthesis [2, 3]. The *de novo* pathway synthesizes new purine nucleotides by building the purine base onto the ribose ring. The pathway starts from phosphoribosyl pyrophosphate (PRPP) and generates inosine monophosphate (IMP) which is then converted into either GMP or AMP depending on the needs of the cell. The *de novo* pathway has provided opportunities for the development of both anticancer that target the folate utilizing enzymes in the pathway [4–10].

**Figure 1:**
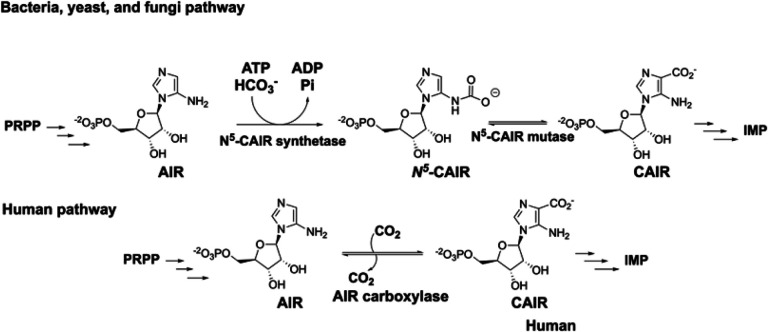
Divergence in purine biosynthesis between bacteria, yeast, and fungi, and humans.

Interestingly, the de novo pathway is different between vertebrates, and lower eukaryotes (yeast and fungi) and bacteria [11] [12–14]. This difference is centered on the conversion of 5-aminoimidazole ribonucleotide (AIR) to 4-carboxy-5-aminoimidazole ribonucleotide (CAIR) (Fig. 1).

Microbes utilize two unique enzymes for this conversion; however, the pathway in vertebrates requires a single enzyme to accomplish the same transformation. In bacteria, yeast and fungi, AIR is converted to the unstable intermediate N^5^-carboxyaminoimidazole ribonucleotide (N^5^-CAIR) by the enzyme N^5^-CAIR synthetase (PurK). PurK carboxylates AIR at the N^5^ position using ATP and bicarbonate as the one- carbon source. N^5^-CAIR is then converted into CAIR by the second enzyme, N^5^-carboxyaminoimidazole ribonucleotide mutase (PurE). In vertebrates, phosphoribosylaminoimidazole carboxylase (AIR carboxylase), an enzyme structurally related to the N^5^-CAIR mutase, directly carboxylates AIR using CO_2_ at the C4 position. No energy is required for this transformation.

The differences between microbes and humans in the conversion of AIR to CAIR provide a biochemical rationale for identifying inhibitors of N^5^-CAIR mutase as potential antibacterial or antifungal agents [13, 15], [16, 17]. Drug discovery efforts focused on N^5^-CAIR mutase have included fragment, small molecules, and natural products using a variety of assays [18–20]. In addition, N^5^-CAIR mutase from different organisms such as *Escherichia coli, Bacillus anthracis, Burkholderia cenocepacia, Legionella pneumophila, and Pyrococcus horikoshii* have been used [19–22]. Sadly, there has not been a single drug-like molecule identified that exhibits potency and selectivity for N^5^-CAIR mutase. The most potent inhibitor, 4-nitro-5-aminoimidazole ribonucleotide (NAIR) was discovered in the 1990’s and has a K_i_ of 0.5 μM for the bacterial enzyme but a K_i_ of 0.34 nM for *Gallus gallus* AIR carboxylase [14]. Thus, NAIR is more potent for the vertebrate enzyme (and likely human AIR carboxylase) than bacterial N^5^-CAIR mutase. In addition, NAIR has troubling physicochemical properties which render the molecule a poor lead agent. Investigations of related azole nucleotide analogs found that even minor changes to NAIR caused diminished inhibition when tested with *G. gallus* AIR carboxylase [14, 23].

To date, high-throughput screening has failed to identify potent and selective drug-like inhibitors of N^5^-CAIR mutase. Fragment-based drug discovery efforts have shown benefit in identifying inhibitors against challenging targets. Fragment screening was conducted to identify binders to *B. anthracis* N^5^-CAIR mutase and a set of 13 fragments with binding affinities ranging from 14 μM to 700 μM were identi ed [20]. Upon validation in an enzyme activity assay, two fragments exhibited modest inhibitory activity [20]. The Firestine lab tested several of the compounds against *E. coli* N^5^-CAIR mutase and found no inhibition suggesting that these compounds are either weak inhibitors of the *E. coli* enzyme or selective inhibitors of *B. anthracis* N^5^-CAIR mutase (unpublished data). Given the previous failures to identify inhibitors against *E. coli* N^5^-CAIR mutase, we elected to conduct a fragment-based screen using a thermal binding assay to identify potential binding fragments. The results of this screen are described below.

## Results

### Thermal Binding Assay for E. coli N^5^-CAIR mutase

**Figure 2:**
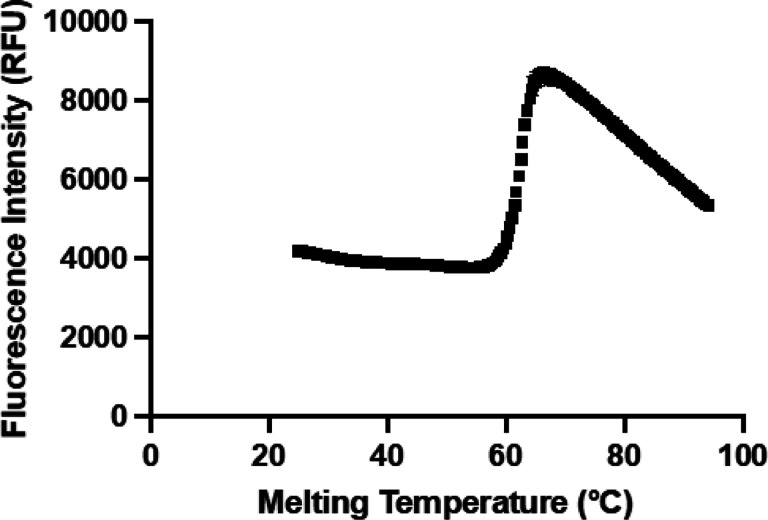
Thermal melting curve of E. coli N^5^-CAIR mutase. Average of duplicates is shown in the graph. Error bars are too small to be visible.

Fragment screening requires a sensitive assay to detect potentially weak binders [24]. One assay that has been extensively used in fragment screens is the thermal shift assay which measures ligand binding based upon a change (either increase or decrease) in the melting temperature of the target. Previously, we developed a thermal shift assay as an orthogonal method to validate hits against N^5^-CAIR mutase and other groups have used it to screen for *B. anthracis* N^5^-CAIR mutase binders [18, 19]. A typical melting curve for N^5^-CAIR mutase is shown in Fig. 2. Replication of the melting curve followed by fitting the complete (25°C to 95°C) curve to the Boltzmann sigmoidal equation (Eq. 1, see methods) gave a melting temperature of 61 ± 0.17°C. This indicates that *E. coli* N^5^-CAIR mutase is highly stable under the assay conditions. The sharp decrease in fluorescence starting at approximately 70°C is well known in thermal shift assays and is due to the precipitation of the unfolded protein/dye complex. Truncation of the curve at 75°C to remove the aggregation phase followed by refitting to Eq. 1 gave a melting temperature that remained unchanged, but the standard error was smaller (61 ± 0.05°C). Given this, subsequent data was truncated to remove the aggregation phase.

**Figure 3:**
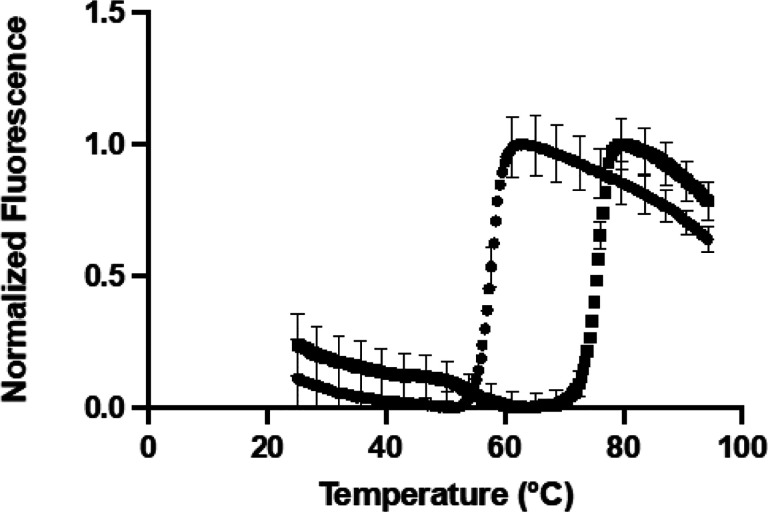
Calculation of Z’. Averages of eight replicates each of thermal melting curves with (■) and without (●) NAIR.

To assess the reproducibility of the assay, the Z’ value was determined (Fig. 3). This metric is commonly used in high-throughput screening campaigns to assess the fitness of the assay for screening [25]. NAIR (20 μM) was used as a positive control since it gave a substantial shift in the melting temperature (ΔT_m_ = 14°C). The negative control was N^5^-CAIR mutase alone. The T_m_ was calculated by fitting all replicates to Eq. 1 to give eight individual melting temperatures that where averaged and the standard deviations calculated. The average melting temperatures were 57 ± 0.36°C for N^5^-CAIR mutase alone and 75 ± 0.32°C for NAIR bound to N^5^-CAIR mutase. The Z’ was calculated to be 0.89 (Eq. 3), indicating that the assay is acceptable for screening, is highly reproducible, and can readily distinguish between compounds that bind versus those that do not [25].

#### Fragment Screen

**Figure 4:**
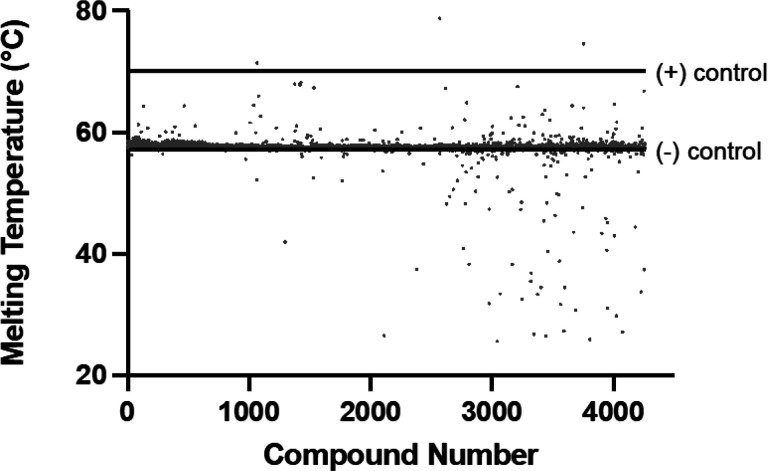
Fragment-screening campaign. A fragment library containing 4,500 compounds was screened for binders of N^5^-CAIR mutase. The average melting temperature of the positive controls are indicated by the line at the top of the graph, while average melting temperature of the negative controls are indicated by the bottom line. Individual melting temperature values for the positive and negative control are not shown for clarity. Each dot represents the melting temperature in the presence of a single compound.

A fragment screen was conducted at the University of Michigan’s Center for Chemical Genomics (CCG) after translation of the thermal shift assay to a 384-well format. Fragments were tested at a concentration of either 250 μM or 500 μM depending on the supplier concentration and the solubility of the fragment. NAIR was used as the positive control and N^5^-CAIR mutase alone was used as the negative control. Each plate contained eight positive control and eight negative control wells. The fragment library consisted of 4,500 compounds taken from Asinex, ChemDiv, Life Chemicals, and PharmBlock. The melting temperatures of N^5^-CAIR mutase with each compound was calculated by fitting the melting curves to Eq. 1. A graph of the campaign was generated by plotting calculated T_m_ values versus compound number (Fig. 4).

We elected to only focus on compounds which stabilized the enzyme since destabilizing compounds do so by binding to the unfolded protein. From the primary screen, 68 compounds showing a temperature increase of 10% or more were selected to be retested (n = 4) at the same concentrations as in the primary assay. Thirty-four fragments showed changes in melting temperature in at least 3/5 assays^[1]^ with > 10% change in melting temperature; most fragments changed protein melting in all assays. Dose- dependent changes in the melting temperature were assessed, in duplicate, for each of the 34 fragments using 8 different concentrations of ligand (see supplemental information for dose-response curves). K_d_ values were calculated using Eq. 2 and are shown in Table 1 [26].

Of the 34 compounds tested, 6 failed to display a dose-response relationship or the K_d_ could not be calculated due to abnormalities in the dose-response curve. The K_d_ values for the fragments ranged from 36 μM to over 1,000 μM with Hill slopes ranging from 1.2 to 38. Abnormally high Hill slopes indicate potential problems with compound aggregation, leading to the identification of false positives [27]. While most investigators prefer Hill slopes close to 1, N^5^-CAIR mutase is an octamer and therefore, the Hill slope can, under certain circumstances, indicate the number of binding sites on the protein.

#### Validation of Dose-Dependent Fragments

To validate these binders, we acquired commercially available compounds to retest binding in the thermal shift assay. A total of 14 fragments were acquired from commercial sources while the remaining fragments were not available at the time of acquisition. The 14 compounds were assessed in duplicate for changes in the melting temperature at a concentration equal to the K_d_ value shown in Table 1. Of the 14 fragments, six showed melting temperature stabilization of N^5^-CAIR mutase while the remaining eight fragments did not. The 6 fragments were subjected to dose-dependence studies and the K_d_ and Hill slope for each fragment is given in Table 2. Compound **26** did not give a suitable dose-response curve, and the resulting K_d_ value displayed a high error. No further studies were done with **26.**

The compounds display differences in K_d_ values between freshly acquired compounds versus those from the screening library with K_d_ values being lower for the newly obtained compounds. We speculate that the observed differences are likely due to some decomposition of the compounds in the screening library rendering the concentration lower than expected or an alteration is the composition of the compounds.

#### Inhibition data from CAIR Decarboxylation Assay

The biophysical data indicates that these compounds bind to N^5^-CAIR mutase and stabilize the protein structure; however, this does not indicate whether binding affects enzyme activity. To assess the effects of these compounds on enzyme activity, inhibition studies were conducted using the CAIR decarboxylation assay. Since this assay measures decarboxylation at 260 nm, only a limited set of concentrations of the fragment could be used in the assay. Dose-dependent inhibition of CAIR decarboxylation was conducted at eight CAIR concentrations ranging from 20 μM to 250 μM using 50 μM and 100 μM concentrations of fragment. For fragment **2**, three concentrations (25, 50, and 150 μM) were evaluated since there was a unique trend that was observed specific to this molecule.

**Figure 5:**
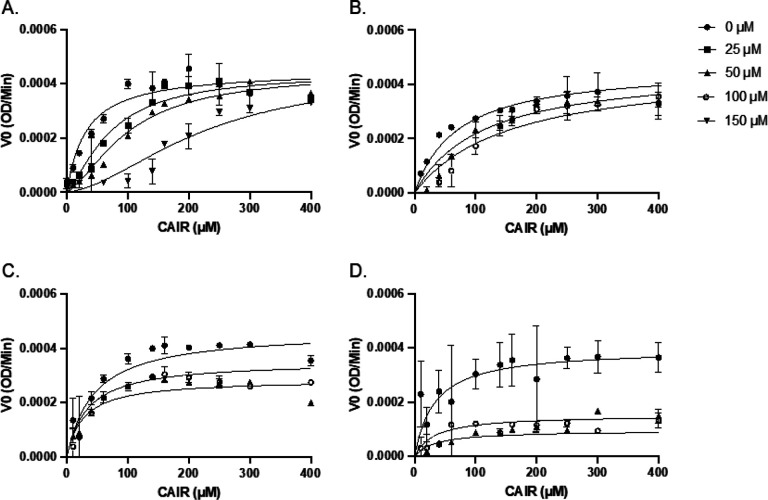
Inhibition of N^5^-CAIR mutase by fragments. **A.** Michaelis-Menten curves for fragment **2** (● 0 μM, ■ 25 μM, ▲ 50 μM, ▼ 150 μM). For the data in the presence of the fragment, the lines shown are for fitting the data to eq. 10. **B.** Michaelis-Menten curves for fragment **9** (● 0 μM, ▲ 50 μM, ∘ 100 μM). For the data in the presence of the fragment, the lines shown are for fitting the data for competitive inhibition (eq. 6). **C.** Michaelis-Menten curves for fragment **22** (● 0 μM, ▲ 50 μM, ∘ 100 μM). For the data in the presence of the fragment, the lines shown are for fitting the data for uncompetitive inhibition (eq. 8). **D.** Michaelis- Menten curves for fragment **25** (● 0 μM, ▲ 50 μM, ∘ 100 μM). for the data in the presence of the fragment, the lines shown are for fitting the data for non-competitive inhibition (eq. 7). For all curves, the data shown in the absence of fragment was fit to the Michaelis-Menten equation (eq. 5).

Inhibition data for the ve fragments is shown in Fig. 5. Calculated V_0_ values from each experiment were plotted against CAIR concentration and the curves were fit to a mixed-model inhibition equation (Eq. 9). The alpha value for each indicated the mechanism of inhibition for each fragment [28]. The curves were then refit to equations applicable to the mechanism of inhibition. For **2**, the curves did not follow standard Michaelis-Menten kinetics and therefore were treated differently. The calculated K_i_ values for each inhibitor are shown in Table 3.

Compound **10** showed no inhibition at the concentrations tested. Compounds **22** (Fig. 5C) and **25** (Fig. 5D) are uncompetitive and non-competitive inhibitors, respectfully, with K_i_ values of 159 μM and 31 μM each. Compound **9** (Fig. 5B) is a competitive inhibitor with a K_i_ value of 83 μM. Compound **2** (Fig. 5A) showed an unexpected sigmoidal Michaelis-Menten curve. Sigmoidal Michaelis-Menten curves can indicate allosteric inhibition; however, no allosteric behavior or binding site has previously been detected on N^5^-CAIR mutase. Sigmoidal curves can also be due to inhibition of substrate binding to other sites on the enzyme (like subunits) by a competitive inhibitor in a highly cooperative manner. The inhibition data for **2** could be fit to Eq. 10, which describes this type of inhibition, with R values ranging from 0.96–0.98. The resulting K_i_ of 4.8 μM is close to its K_d_ value and indicates that **2** is a highly potent fragment.

## Discussion

Here, we report a fragment screen of 4,500 commercially available fragments against *E. coli* N^5^-CAIR mutase. Ultimately, four compounds displayed dose-dependent binding to and inhibition of the enzyme. The fragments displayed K_d_ values ranging from 9–309 μM with K_i_ values from 4–159 μM.

Compound **2** was the most potent fragment and showed inhibition of N^5^-CAIR mutase, but with a sigmoidal Michaelis-Menten plot. The sigmoidal curve usually suggests allosteric behavior; however, no reported allosteric regulators of N^5^-CAIR mutase have been reported. N^5^-CAIR mutase exists as a homo octameric protein in which the active sites of each monomer are close to each other. It is possible that the binding of **2** alters the binding of substrate to another active site. Kinetic analysis has shown that competitive inhibitors that bind to one site in a multisite system but prevent binding of a substrate to another site will result in a sigmoidal Michaelis-Menten plot. The data for **2** fits well to this model. Such an analysis suggests that there is “communication” between active sites or that **2** can occupy both active sites simultaneously. However, there is no additional information that would support occupancy at two sites. Ultimately, the determination of a crystal structure for N^5^-CAIR mutase with **2** would be highly informative to determine how **2** binds to the enzyme and whether any changes in the conformation of the enzyme occur upon binding.

An examination of the Hill slope of the binding curves provides support for cooperativity between binding site of the inhibitor. While Hill slope is normally used as a measure of cooperativity, it has also been used as the number of binding sites for the inhibitor. The latter is only true if there is strong cooperativity between the binding sites. Interestingly, N^5^-CAIR mutase is an octamer made up of eight identical subunits and theoretically 8 binding sites. Given the fact that most of the Hill slopes are around 4, it is tempting to speculate that the compounds are binding to 4 out of the 8 sites and may be doing so in a highly cooperative manner. In support of this, the crystal structure of *E. coli* N^5^-CAIR mutase with CAIR showed higher occupancy of a nucleotide at 4 out of the 8 active sites. In contrast, the structure of NAIR bound to *E. coli* N^5^-CAIR mutase shows this inhibitor bound to all eight active sites. Ultimately, additional biochemical or structural studies are needed to determine the number of inhibitors bound per octamer.

**Figure 6:**
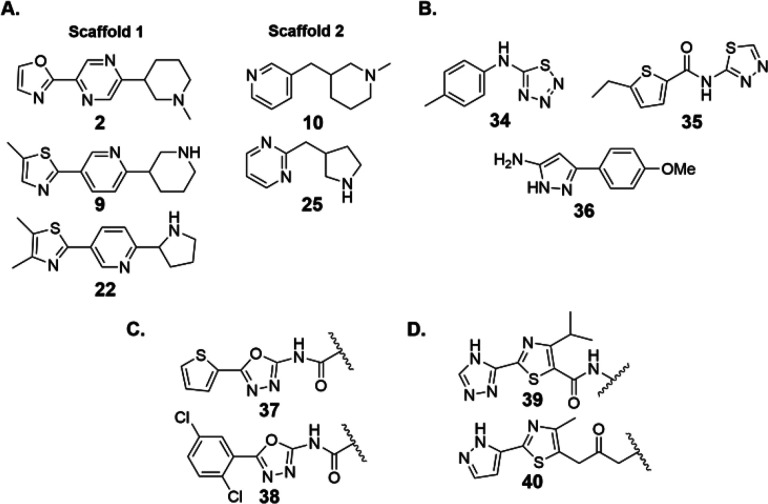
Compounds reported to bind to N^5^-CAIR mutase or PAICS. **A.** Fragments identified from this work can be classified into two scaffolds. Scaffold 1 fragments containing a 5-member heterocycle connected to either a piperidine or pyrrolidine substituted pyridine or pyrazine ring. Scaffold 2 fragments contain a piperidine or pyrrolidine heterocycle connected to a 6-membered aromatic heterocycle. **B.** Fragments identified that bind to B. anthracis N^5^-CAIR mutase. **C.** Compounds identified from high-throughput screening reported to bind to B. anthracis N^5^-CAIR mutase. The squiggly line indicates the site of attachment to other part of the molecule not shown. **D.** Fragments identified in a recent proteomics study reported to binding to human PAICS protein. The squiggly line represents the site of attachment to a photocrosslinker used in the study.

The identified fragments could be clustered into two scaffolds (Fig. 6A). Compounds **2, 9** and **22** contain a 5-member heterocycle connected to either a piperidine- or pyrrolidine-substituted pyridine or pyrazine ring. The second scaffold, composed of **10** and **25** contains a piperidine or pyrrolidine heterocycle connected via a one carbon linker to a 6-membered aromatic heterocycle. Interestingly, the compounds identified in this paper are structurally related to compounds previously identified against *B. anthracis* enzyme. As shown in Fig. 6B and C, previous fragment (Fig. 6B) or high-throughput (Fig. 6C) screens identified five membered heterocycles attached to aromatic rings. Two fragments that both bound and inhibited activity (**34, 35**) show similarity to scaffold 2. Despite the similarity to scaffold 2, we have found that **34** and **35** do not show inhibition of *E. coli* N^5^-CAIR mutase up to a concentration of 100 μM (data not shown) suggesting that these compounds are either weaker inhibitors of the *E. coli* enzyme or are selective for the *B. anthracis* N^5^-CAIR mutase. Fragment **36** is a modest binder to *B. anthracis* enzyme and is related to scaffold 1. Compounds **37** and **38**, which were identified by high-throughput screening, both bound to and inhibited *B. anthracis* N^5^-CAIR mutase. These compounds are similar to scaffold 1. Whether **36, 37** or **38** bind to *E. coli* N^5^-CAIR mutase remains to be determined.

A significant challenge in the field is developing selective inhibitors for N^5^-CAIR mutase since there is an evolutionary and structural relationship between human AIR carboxylase and the bacterial enzyme [4, 14]. Despite these similarities, the 1000-fold selectivity of NAIR for AIR carboxylase over N^5^-CAIR mutase indicates that it is possible to specifically inhibit one of these enzymes over the other. To date, there have been no inhibitors that have shown significant selectivity for the N^5^-CAIR mutase over AIR carboxylase, and we have not addressed this issue in this publication. However, data in a recent publication suggests that these compounds are likely to inhibit both AIR carboxylase and N^5^-CAIR mutase. A broad, quantitative proteomics study was conducted to identify novel protein-ligand interactions between a collection of over 400 fragments and the human proteome. This work discovered a set of three fragments that bound to the human PAICS protein [29]. Two of these fragments (**39, 40**) are shown in Fig. 6D and an examination reveals that they are structurally related to **2**. The fact that similar fragments are identified via two distinctly different approaches suggests that these fragments are likely legitimate binders of AIR carboxylase and/or N^5^-CAIR mutase. Additional medicinal chemistry studies are needed to determine whether **39** and **40** bind to N^5^-CAIR mutase and/or AIR carboxylase and whether related fragments uncovered here are selective or general inhibitors of these enzymes. This work will be reported in due course.

## Materials and Methods

### General.

Unless otherwise stated, all chemicals, cells and reagents were obtained from ThermoFisher Scientific or Sigma. Plasmid pJK057 was obtained from Addgene. NAIR and CAIR were prepared according to the literature [13, 23, 30, 31]. Cobalt resin was obtained from Gold Biotechnology. The fragment-based screen was conducted at the University of Michigan’s Center for Chemical Genomics. The fragment library used in the screen is a bespoke collection of fragments obtained from commercial vendors and assembled by the staff at the Center for Chemical Genomics. Fragments were purchased from Asinex or Life Chemicals and were used as obtained. All fragments were dissolved into DMSO before use.

#### Purification of E. coli N^5^-CAIR mutase

The plasmid pJK057 was transformed into BL21(DE3) and plated onto LB-Amp (100 μg/mL) plates and grown overnight at 37°C. One liter of LB media containing 100 μg/mL ampicillin was inoculated with 10 mL of an overnight starter culture of transformed BL21(DE3) and the culture grown with shaking at 250 RPM at 37°C until the OD_600_ was between 0.6–0.8. Protein expression was initiated by the addition of 1 mM IPTG, and the temperature was decreased to 18°C. The culture was shaken overnight at 18°C, and the cells harvested by centrifugation at 4°C. The resulting cell pellet was stored at −80°C until use.

A high-density cobalt agarose column (5 mL) was prepared by washing with distilled water followed by equilibrating with 10 column volumes of buffer 1 (50 mM sodium phosphate, 300 mM NaCl, pH 7.4) containing 10 mM imidazole. The cell pellet was thawed on ice and treated with Bacterial Protein Extraction Reagent (ThermoFisher Scientific) according to the manufacturer’s instructions. After incubating on ice for 10–20 minutes, the lysed bacteria were centrifuged at 4°C at 16,000 RPM for 30 minutes. The lysate was treated to a final concentration of 1% streptomycin sulfate to remove nucleic acids, and the resulting cloudy solution was centrifuged at 4°C at 16,000 RPM for 30 minutes. The lysate was added to the freshly prepared column and unbound material was removed by washing with 10 column volumes of buffer 1 containing 25 mM imidazole followed by 10 column volumes of buffer 1 containing 50 mM imidazole. N^5^-CAIR mutase was eluted from the column using a step gradient of 15 mL of buffer 1 containing 150 mM imidazole to 15 mL of buffer 1 containing 400 mM of imidazole. Individual fractions (1.5 mL) were analyzed by SDS-PAGE to identify those containing N^5^-CAIR mutase. These fractions were combined and dialyzed (10K MWCO, Thermo Scientific Slide-A-Lyzer^™^ dialysis cassette) at 4°C in 10 mM Tris, 200 mM NaCl, pH 8 storage buffer. The protein was concentrated by centrifugal concentration using a 10K MWCO Amicon Ultra centrifugal lter. Protein (concentration ranged from 5 mg/mL to 15 mg/mL) was stored as 10 μL aliquots at −80°C until use. The protein was judged to be at least 95% pure based upon an overloaded SDS-PAGE gel.

#### Thermal Shift Assay

This assay was conducted using Temp-Plate 96-well no-skirt 0.2 mL PCR plates (USA Scientific). For the positive control wells, each well contained a final volume of 10 μL with 25 mM Tris (pH 8), 20 μM NAIR, 5 μM N^5^-CAIR mutase (concentration determining using monomer weight), 10x SYPRO Orange (Invitrogen), and 0.5 μL DMSO. Negative control wells contained the same components but lacked NAIR. Plates were sealed with TempPlate RT select optical film (USA Scientific) and spun down for one minute prior to running on a Stratagene Mx3005P QPCR system with a temperature range between 25°C and 95°C over 40 minutes. The resulting melting curves were imported into GraphPad Prism 10 and fitted to the Boltzmann-sigmoidal equation (Eq. 1) after truncation to remove the aggregation phase of the curve. For dose-dependent studies, the resulting T_m_ values were t to Eq. 2. For experiments examining binding of fragments, 0.5 μL of fragment from the appropriate stock concentration dissolved in DMSO was added to each well along with the components from the negative control. The melting experiment was conducted and analyzed as stated above.

#### Analysis of Melting Curve Data

The thermal melting curves were fit to the Boltzmann Eq. (1) to determine the melting temperature [32, 33]. In the equation, *Y* = = fluorescence intensity; *X* = temperature; *Top* and *Bottom* represent maximal fluorescence at top of curve and baseline fluorescence at bottom of curve, respectively; *T*_*m*_ = protein melting temperature; *Slope* represents steepness of curve.

(1) $$Y=\text{Bottom}+\frac{\text{Top}-\text{Bottom}}{1+\text{exp}\left(T_{m}-\frac{X}{\text{Slope}}\right)}$$

For dose-response studies, each curve was fit according to Eq. 1 to generate the melting temperature. A plot of melting temperature versus compound concentration was generated and the resulting data was fit to a four-parameter non-linear regression Eq. (2) where *Y* is the response, *Top* and *Bottom* represent top and bottom of plateau in the same units as *Y* respectively, *K*_*d*_ is the dissociation constant in the same units as *X* and *Hill slope* is unitless and describes the steepness of the curve.

(2) $$Y=\text{Bottom}+\frac{\text{Top}-\text{Bottom}}{{\left(1+\frac{K_{d}}{X}\right)}^{\left(\text{HillSlope}\right)}}$$

To calculate the robustness of the assay, the Z’ value was calculated using Eq. 3 where the variables μ*p* is the mean of all positive controls, *μn* is the mean of all negative controls, *σp* is the standard deviation of all positive control and *σn* is the standard deviation of all negative controls.

(3) $$Z^{\prime }=1-\frac{3(\sigma \;p+\sigma \;n)}{\left|\mu \;p-\mu \;n\right|}$$

### Fragment-Based Screening Using Thermal Shift Assay.

For the primary screen and single concentration validation studies (n = 4) each assay plate was set-up as follows: In an Applied Biosystems^™^ MicroAmp^™^ Optical 384-well reaction plate, eight positive control wells contained 25 mM Tris, pH 8, 20 μM NAIR, 5 μM N^5^-CAIR mutase, and 10x SYPRO orange protein gel stain (Invitrogen). Negative control wells contained the same components but lacked NAIR. All wells contained a final volume of 10 μL. For sample wells, fragments were dispensed into the wells at a final concentration of either 250 μM or 500 μM depending on the concentration of the stock plate, using an Echo liquid handling system. Buffer was added, followed by SYPRO dye and N^5^-CAIR mutase. Plates were sealed with film and spun down for one minute at 1,000 RPM prior to running the thermal binding experiment in a QuantStudio^™^ 7 Flex real-time PCR system using conditions stated in the melting curve experiment. The melting temperature range for each fragment was between 25°C to 95°C. The thermal melting curves were fit to the Boltzmann Eq. (1) to determine the melting temperature [32, 33].

For dose-dependence studies, eight concentrations for each fragment were tested in duplicate. The concentrations tested were 1,000, 690, 475, 327, 225, 155, 107, and 75 μM or 500, 345, 238, 164, 113, 78, 53, and 37 μM depending on the concentration of the stock plates which were either at 100 mM or 50 mM. For dose-dependence studies, K_d_ values were calculated according to Eq. 2.

#### Activity Assay

Cuvette Method. In a quartz cuvette with a final volume of 1 mL, 975 μL 25 mM Tris (pH 8 + .01% triton) buffer was added along with either 5 μL DMSO or 5 μL of compound dissolved in DMSO, and 10 μL CAIR from appropriate stocks ranging from 0.5 mM and 25 mM. The reaction was initiated by the addition of 24 nM N^5^-CAIR mutase, and the reaction was monitored using a Cary 1 spectrophotometer at 260 nm over time. The initial velocity was calculated for the first minute of the enzymatic reaction.

### Microplate Method

In a non-sterile 96-well at bottom Corning^®^ UV-transparent microplate, each well had a final volume of 100 μL and contained 83 μL 25 mM Tris (pH 8 + .01% triton), 5 μL CAIR from stocks ranging from 0.2 mM and 8 mM, and either 2 μL DMSO or 2 μL compound dissolved in DMSO. The reaction was initiated by the addition of 24 nM N^5^-CAIR mutase, and the progress of the enzymatic reaction was monitored over 40 minutes using a Biotek Synergy 2 multimode plate reader. Progress curves were imported into Excel and normalized to zero absorbance at time zero. The resulting curves were fitted to Eq. (4) using KaleidaGraph to determine the initial velocity (V_0_). For Eq. 4,

(4) $$P=\frac{V_{0}}{\eta }\left(1-e^{-\eta \;t}\right)$$

*P* represents product formation, *V*_*0*_ represents the initial velocity, and *h* is the rate of change for the non-linear portion of the plot [34]. After calculating the V_0_, the data was imported into GraphPad Prism 10, and the resulting plot of V_0_ versus CAIR was fitted to the Michaelis-Menten Eq. (5) or equations 6–10 to determine the inhibition value and mechanism of inhibition.

#### Michaelis-Menten:


(5) $$v_{o}=\frac{V_{\text{max}}*S}{K_{m}+S}$$

e variables are defined as: v_0_=initial velocity; V_max_ = maximum enzyme velocity; K_m_ =Michaelis-Menten constant; S=substrate concentration.

#### Competitive inhibition:


6 $$v_{0}=\frac{V_{\text{max}}*S}{K_{\text{mobs}}+S}$$

$$K_{\text{mobs}}=K_{m}*\left(1+\frac{\left[I\right]}{K_{i}}\right)$$

The variables are defined as: v_0_ = initial velocity; I = inhibitor concentration; K_i_ = inhibition constant; V_max_ = maximum enzyme velocity; K_m_ = Michaelis-Menten constant; S = substrate concentration.

#### Noncompetitive inhibition:


(7) $$v_{0}=V_{\text{maxinh}}*S/〖\left(K{〗}_{m}+S\right)$$

$$V_{\text{maxinh}}=V_{\text{max}/\left(1+I/K_{i}\right)}$$

The variables are defined as: v_0_ = initial velocity; I = inhibitor concentration; V_max_ = maximum velocity in the absence of an inhibitor; K_m_ = Michaelis-Menten constant in the absence of an inhibitor; K_i_ = inhibition constant; S = substrate concentration.

#### Uncompetitive inhibition:


8 $$v_{0}=V_{\text{maxApp}}*S/\left(K_{\text{mApp}}+S\right)$$

$$V_{\text{maxApp}}=V_{\text{max}/\left(1+I/K_{i}\right)}\;K_{\text{mApp}}=K_{m}/\left(1+I/K_{i}\right)$$

The variables are defined as: v_0_ = initial velocity; I = inhibitor concentration; V_max_ = maximum velocity in the absence of an inhibitor; K_m_ = Michaelis-Menten constant in the absence of an inhibitor; K_i_ = inhibition constant; S = substrate concentration.

#### Mixed-model inhibition:


9 $$v_{0}=V_{\text{maxApp}}*S/\left(K_{\text{mApp}}+S\right)$$

$V_{\text{maxApp}}=\frac{V_{\text{max}}}{1+\frac{I}{\text{Alpha}*K_{i}}}\;K_{\text{mApp}}=K_{m}*\frac{1+I/K_{i}}{1+\frac{I}{\text{Alpha}*K_{i}}}$ The variables are defined as: v_0_=initial velocity; I = inhibitor concentration; V_max_= maximum velocity in the absence of an inhibitor; K_m_ = Michaelis-Menten constant in the absence of an inhibitor; K_i_ = inhibition constant; Alpha = determines the mechanism.

#### Competitive Inhibition in Multisite Systems


(10) $$v_{0}=\frac{V\text{max}*\left(\frac{S}{K_{m}}+\frac{S^{2}}{{K_{m}}^{2}}\right)}{1+2\frac{S}{K_{m}}+\frac{S^{2}}{{K_{m}}^{2}}+\frac{I}{K_{i}}}$$

The variables are defined as: v_0_ = initial velocity; V_max_ = maximum enzyme velocity; K_m_ = Michaelis-Menten constant; K_i_=inhibition constant; S = substrate concentration.

## Footnotes

1. The five total assays include the primary assay in addition to the secondary assay conducted with four replicates.
